# Identification of Candidate MYB Regulators of Seed Coat Color and Oil Content in *Brassica napus*

**DOI:** 10.3390/ijms27209161

**Published:** 2026-10-15

**Authors:** Liwei Zheng, Xu Gao, Fan Wang, Xue Zhao, Xiaoyang Liu, Lijing Zhang, Jinyong Huang, Hongbo Chao, Weiguo Zhao

**Affiliations:** 1 School of Agricultural and Biomanufacturing, Zhengzhou University, Zhengzhou 450001, China; liwei_zheng@zzu.edu.cn (L.Z.); 13937144297@163.com (X.G.); wangfan992022@163.com (F.W.); xzhaoez@163.com (X.Z.); 19846850473@163.com (X.L.); 2 School of Life Sciences, Zhengzhou University, Zhengzhou 450001, China; 13214469579@163.com (L.Z.); jinyhuang@zzu.edu.cn (J.H.); 3 Xianghu Laboratory, Hangzhou 311231, China; 4 School of Modern Agriculture and Biotechnology, Ankang University, Ankang 725000, China

**Keywords:** proanthocyanidin biosynthesis, flavonoid metabolism, gene co-expression network, natural variation, rapeseed improvement

## Abstract

Yellow-seeded *Brassica napus* varieties are highly desirable for breeding due to their superior oil content and seed quality. Proanthocyanidins (PAs) are the major flavonoids determining seed coat color, which is closely associated with oil accumulation. MYB transcription factors, especially R2R3-MYBs, act as important regulators of flavonoid biosynthesis and multiple developmental processes and may be associated with variation in PA accumulation and oil content. In this study, we performed a genome-wide identification and characterization of PA biosynthesis-related genes and MYB transcription factors in *B. napus*, and a total of 102 PA biosynthesis-related genes (*BnaPABGs*) and 416 high-confidence R2R3-MYBs (*BnaMYBs*) were identified and characterized. Transcriptome profiling of yellow- and black-seeded accessions at distinct developmental stages uncovered divergent expression patterns of both PA-related genes and MYBs. Weighted gene co-expression network analysis (WGCNA) identified a central module that co-enriched the majority of PA biosynthesis-related genes along with numerous lipid metabolism-related genes, suggesting a potential co-expression relationship between flavonoid metabolism and oil accumulation during seed maturation. By integrating co-expression networks with previously reported quantitative trait loci (QTLs) for seed coat color and oil content, we identified candidate *BnaMYBs* potentially associated with seed coat pigmentation and oil accumulation. Among these candidates, *BnaA08.MYB61*, *BnaC06.MYB63*, and *BnaC03.MYB56* were prioritized as candidate genes based on their expression patterns and co-expression relationships, while haplotype analyses provided additional support for *BnaC03.MYB56* and *BnaA08.MYB61*. Haplotype analyses further revealed supported associations between natural variation at *BnaC03.MYB56* and *BnaA08.MYB61* loci and seed oil content, whereas *BnaC06.MYB63* showed limited haplotype diversity. Collectively, our findings provide new insights into the potential relationship of flavonoid and lipid metabolism in *B. napus* seeds and provide candidate genetic resources for further functional investigation of seed coat pigmentation and oil accumulation in *B. napus*.

## 1. Introduction

Rapeseed (*Brassica napus* L., AACC, 2n = 38) is one of the most important oil crops worldwide and serves as a major source of edible vegetable oil. As an allotetraploid species, *B. napus* originated from the natural hybridization between *B. rapa* (AA, 2n = 20) and *B. oleracea* (CC, 2n = 18) approximately 7500 years ago, followed by extensive domestication and genetic improvement [1,2]. Due to its considerable economic value, improving seed oil content has remained a major objective in rapeseed breeding programs. Therefore, identifying genes associated with important quality traits is crucial for understanding their genetic basis and providing valuable candidate resources for molecular breeding.

Seed coat color is an important agronomic trait and is closely associated with seed quality traits, including oil content, protein content, and overall economic value of the rapeseed products [3]. In *B. napus*, yellow-seeded lines typically exhibit higher oil content, thinner seed coats, and reduced accumulation of fiber-related compounds compared with black-seeded lines [4]. The consistent phenotypic association was frequently found between seed coat color and oil content, which indicates a phenotypic relationship between these traits. Previous QTL studies have further revealed overlapping genomic regions associated with seed coat color and oil content; however, whether these shared regions contain common functional genes remains unclear [5,6,7,8]. Seed coat pigmentation is primarily determined by the accumulation of proanthocyanidins (PAs), the major flavonoid compounds deposited in the seed coat [9,10]. As a specialized branch of the flavonoid pathway, PA biosynthesis originates from the general phenylpropanoid metabolism and is orchestrated by a series of structural genes, whose expression is tightly regulated by a suite of regulatory transcription factors [11]. Several flavonoid biosynthetic and regulatory genes, including *TT2*, *TT7*, *TT8*, and *TT18*, have been shown to influence seed coat color and are also associated with variation in seed oil content in rapeseed [12,13]. These findings suggest that flavonoid metabolism may contribute to the association between seed pigmentation and oil accumulation. In addition, several quantitative trait loci (QTLs) associated with seed coat color and oil content have been shown to overlap in rapeseed, providing genetic evidence for the potential relationship between these two traits. Identifying candidate genes within these overlapping regions is therefore important for dissecting the genetic basis underlying the relationship between seed coat pigmentation and oil accumulation [14].

The biosynthesis of flavonoids and PAs is controlled by a complex transcriptional regulatory network, in which members of the R2R3-MYB family play central regulatory roles [15,16,17]. Through interactions with bHLH and WD40 proteins, R2R3-MYB transcription factors regulate the expression of flavonoid biosynthetic genes, thereby controlling PA accumulation and seed coat pigmentation [17,18]. The MYB transcription factor family is among the largest and most functionally diverse transcription factor families in plants, widely involved in regulating growth and development, secondary metabolism, and stress responses [19,20,21]. During seed development, MYB transcription factors play a central regulatory role, governing multiple critical processes from seed morphogenesis to storage reserve accumulation. In rapeseed, several MYB transcription factors have been reported to be associated with seed coat color, oil content, or both [4,22,23]. Whether additional MYB transcription factors are associated with the genetic relationship between seed coat pigmentation and oil content remains to be further explored.

Numerous loci associated with seed coat color and oil content have been identified, and the co-localized QTLs provide valuable targets for identifying genes that may contribute to variation in both traits. Therefore, in the present study, to discover potential MYBs for seed coat color and oil content, we performed genome-wide identification of PA biosynthesis-related genes and MYB transcription factors in the *B. napus* genome. By integrating seed coat color and oil content QTL co-location, mining of candidate genes underlying QTLs, and co-expression analysis of MYB with genes related to PA biosynthesis and oil content, we systematically identified candidate MYBs potentially involved in seed coat pigmentation and oil content. Our findings provide candidate genetic resources for understanding the potential relationship between PA accumulation and oil accumulation in rapeseed and provide a foundation for further functional investigation of yellow-seeded, high-oil-content cultivars.

## 2. Results

### 2.1. Genome-Wide Identification and Evolutionary Analysis of PA Biosynthesis-Related Genes in B. napus Genome

**Figure 1:**
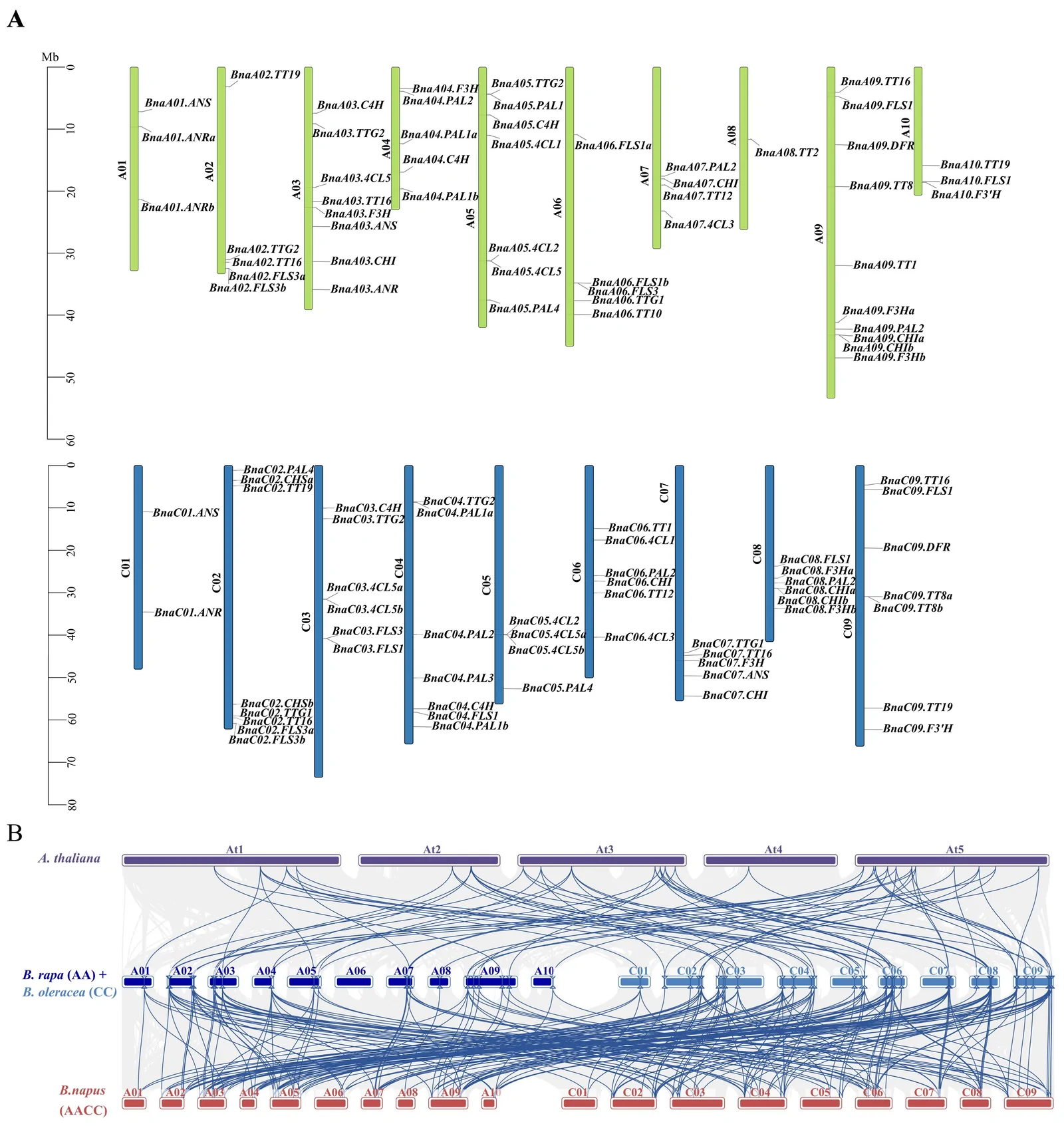
Chromosomal distribution and synteny analysis of proanthocyanidin biosynthesis-related genes in *Brassica napus* and related Brassica species. (**A**) Physical distribution of proanthocyanidin biosynthesis-related genes across the 19 chromosomes of *B. napus*. Green and blue chromosomes represent the A and C subgenomes, respectively. Gene names are indicated adjacent to their corresponding chromosomal positions, which were determined based on physical coordinates. The scale on the left indicates chromosome length in megabases (Mb). (**B**) Syntenic relationships of PA biosynthesis-related genes among *A. thaliana*, *B. rapa* (AA), *B. oleracea* (CC), and *B. napus* (AACC). Background lines represent genome-wide syntenic relationships, whereas highlighted lines indicate syntenic gene pairs associated with proanthocyanidin biosynthesis.

A total of 102 *Brassica napus* genes homologous to 27 *Arabidopsis thaliana* genes involved in proanthocyanidin (PA) biosynthesis were identified, including 76 structural genes, 20 transcription factors, and 6 transporter-related genes (Table S1). The proanthocyanidin biosynthesis-related genes (designated as *BnaPABGs*) were unevenly distributed across the *B. napus* genome, with several duplication events detected across the *B. napus* genome (Figure 1A). Several duplicated gene copies were observed across the *B. napus* genome, including tandemly duplicated *CHI* on chromosome A09 (*BnaA09.CHIa*/*BnaA09.CHIb*), *TT8* on chromosome C09 (*BnaC09.TT8a/BnaC09.TT8b*), *CHI* on chromosome C08 (*BnaC08.CHIa/BnaC08.CHIb*), *4CL* on chromosome C05 (*BnaC05.4CL5a/BnaC05.4CL5b*), FLS3 on chromosome A02 (*BnaA02.FLS3a/BnaA02.FLS3b*), and *FLS3* on chromosome C02 *(BnaC02.FLS3a/BnaC02.FLS3b*). The presence of multiple copies is likely attributable to the allotetraploid origin of *B. napus* and the retention of duplicated genomic regions after whole-genome duplication events. Therefore, duplicated copies derived from A and C subgenomes were considered homoeologous genes, whereas additional duplicated copies generated by local duplication events were classified as paralogous genes. Subgenome classification showed that 50 *BnaPABGs* were retained as syntenic orthologs in the LF, MF1, and MF2 subgenomes, whereas the remaining genes were classified as non-syntenic homologs (Table S1). Comparative synteny analysis revealed conserved evolutionary relationships of PA biosynthesis-related genes among *Arabidopsis thaliana*, *Brassica rapa*, *Brassica oleracea*, and *Brassica napus* (Figure 1B). Numerous orthologous relationships were identified between the A subgenome of *B. napus* and its progenitor *B. rapa*, as well as between the C subgenome and its progenitor *B. oleracea*. In particular, extensive syntenic conservation between *B. oleracea* and the C subgenome indicates substantial retention of PA biosynthesis-related genes after allopolyploidization. Similarly, conserved collinear relationships between the A subgenome and *B. rapa* suggest that both ancestral genomes contributed to the maintenance and diversification of PA biosynthetic pathways. These results demonstrate that PA biosynthesis-related genes have undergone extensive evolutionary retention during the formation and diversification of the allotetraploid *B. napus* genome.

### 2.2. Genome-Wide Identification and Expression Analysis of BnaMYBs in B. napus Seeds

MYB transcription factors have been frequently implicated in the regulation of seed PA deposition and seed development including lipid accumulation in plants [24,25]. As most known MYB regulators of flavonoid and proanthocyanidin biosynthesis belong to the R2R3-MYB subfamily, we focused on this group to identify BnaMYBs potentially associated with PA accumulation in *B. napus*. Firstly, a phylogenetic tree was constructed using *B. napus* R2R3-MYBs (*BnaMYBs*) with conserved MYB domains, *A. thaliana* R2R3-MYBs, and functionally characterized PA-related MYBs from other plant species (Table S2; Figure S1). BnaMYBs phylogenetically close to known flavonoid/PA-related MYBs were considered potential candidates associated with PA biosynthesis and seed coat pigmentation.

Based on the transcriptome data from yellow- and black-seeded *Brassica napus* varieties at 24 and 33 days after flowering (DAF), the expression characteristics of *BnaMYBs* were investigated during seed development in different tissues of seeds [26], especially in the seed coat. Hierarchical clustering analysis (HCA) of BnaMYB expression profiles across the analyzed tissues and developmental conditions (K24_SC, K33_SC, K24_OC, K33_OC, K24_IC, K33_IC, K24_R and K33_R) revealed substantial variation in their expression patterns among different tissues during seed development (Figure S2). A subset of *BnaMYBs* was found to show preferential expression in the seed coat, whereas several genes also displayed distinct expression patterns between the yellow- and black-seeded materials. These genes may therefore be involved in seed coat development, proanthocyanidin accumulation, or pigmentation.

**Figure 2:**
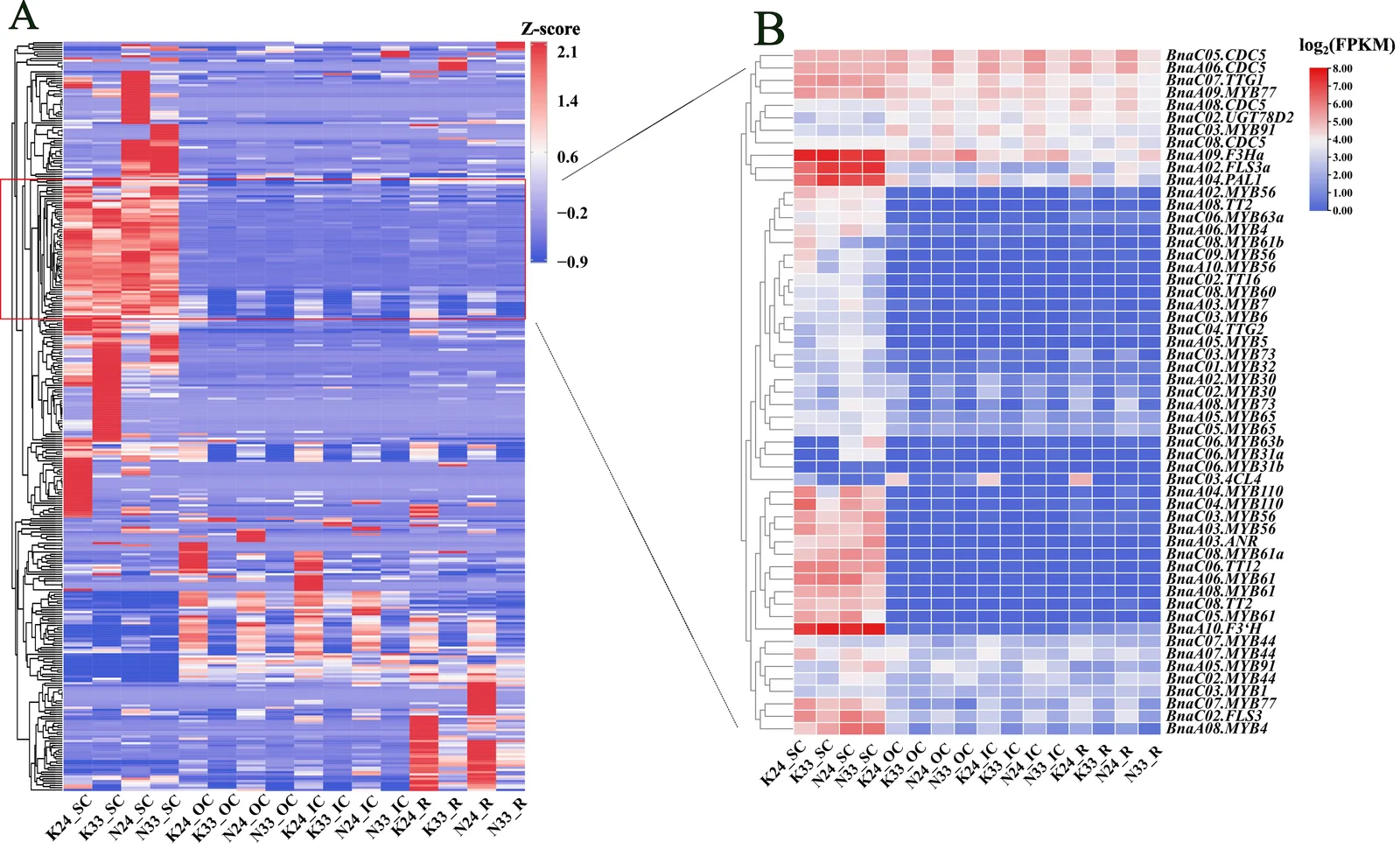
Expression heatmap of proanthocyanidin biosynthesis-related genes and *BnaMYB* genes in *Brassica napus*. (**A**) Hierarchical clustering analysis of *BnaMYB* genes together with proanthocyanidin biosynthesis-related genes (*BnaPABGs*) based on their expression profiles across different seed tissues and developmental stages. Rows represent genes, and columns represent samples from SC, OC, IC and R collected from yellow- and black-seeded materials at 24 and 33 days after flowering (DAF). Genes were hierarchically clustered, whereas samples were displayed in their original order. Expression values were standardized using Z-score transformation. The color gradient represents relative expression levels, with red indicating higher and blue indicating lower expression. (**B**) Expression heatmap of the selected cluster enriched in proanthocyanidin biosynthesis-related genes. The selected cluster contained multiple *BnaPABGs* and *BnaMYBs* showing similar expression patterns, including genes involved in proanthocyanidin biosynthesis and regulation. Rows represent genes and columns represent samples. The color scale represents log_2_(FPKM) values.

To further identify *BnaMYBs* potentially involved in proanthocyanidin biosynthesis, *BnaMYBs* were further analyzed together with identified *BnaPABGs* based on their expression profiles; HCA of the *BnaMYBs* together with identified *BnaPABGs* was implemented (Figure 2A); and the cluster enriched in *BnaPABGs* was highlighted and their expression patterns were visualized separately (Figure 2B). Several important *BnaPABGs*, including *TT2*, *TTG1*, *TT16*, *TT12*, *CHS*, *F3H*, *FLS3*, and *PAL1*, exhibited differential expression patterns between the yellow- and black-seeded varieties in the seed coat. The specific subsets of *BnaMYBs* may be associated with proanthocyanidin accumulation during seed coat development in *B. napus.*

### 2.3. Identification of BnaMYBs Located Within Co-Localized QTL Regions for Seed Coat Color and Oil Content in B. napus

**Figure 3:**
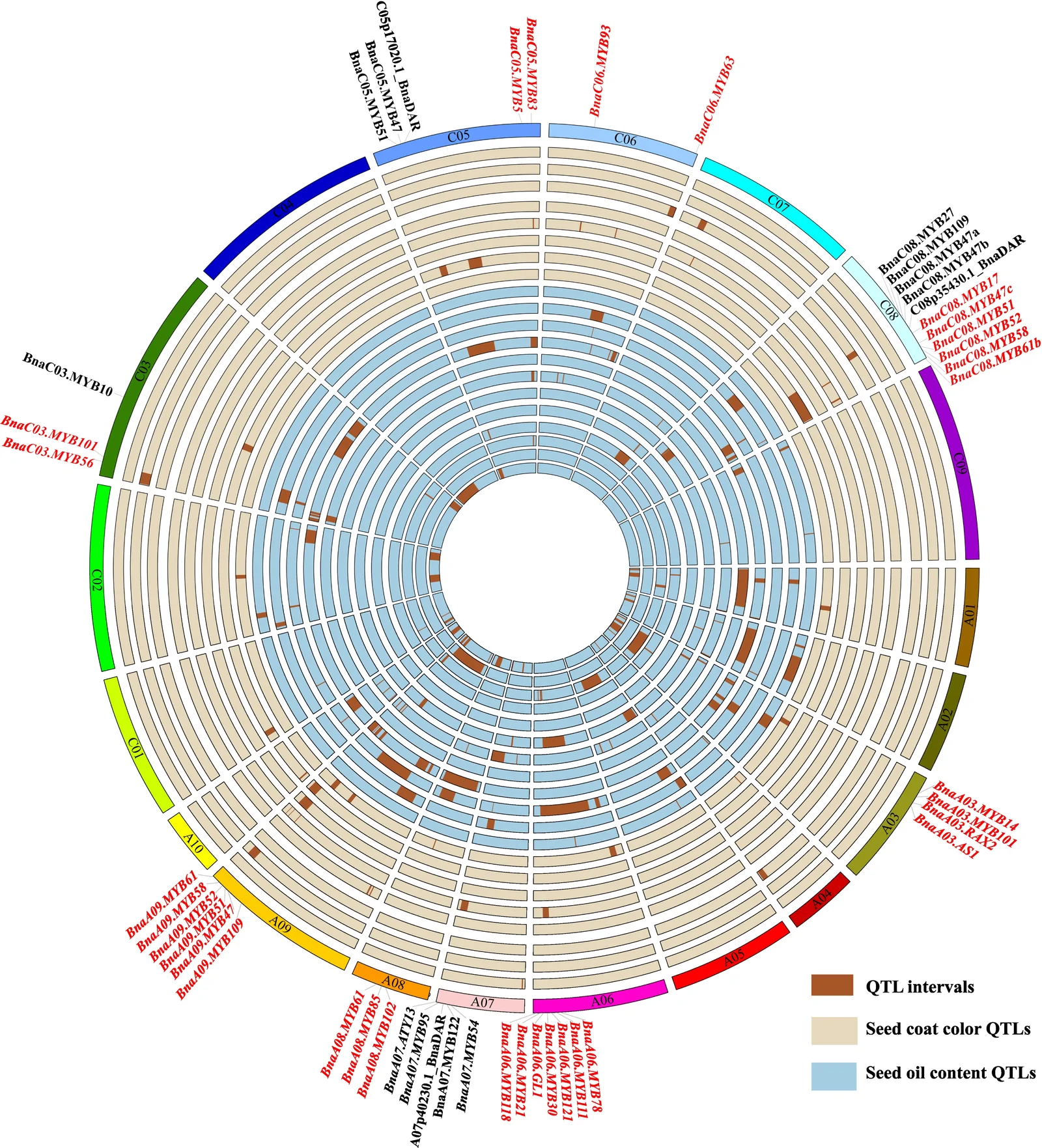
Chromosomal distribution of seed coat color- and oil content-associated QTLs and candidate R2R3-BnaMYB genes in *B. napus*. Brown blocks indicate mapped QTL intervals. Red labels represent *BnaMYBs* located within co-localized QTL regions between seed coat color and oil content traits, whereas black labels represent *BnaMYBs* located within only one type of QTL region. From inside to outside, the 20 inner circles consist of 12 light-brown tracks representing oil content QTL datasets and 8 light-blue tracks representing seed coat color QTL datasets. The short brown bars within these tracks indicate the physical positions of mapped QTL intervals. The blocks at the outermost circle represent the 19 chromosomes of *B. napus*. The gene labels outside the outermost circle show the candidate genes and their physical positions. For visualization clarity, only *BnaMYBs* located within QTL regions co-localized between seed coat color and oil content, as well as those located within seed coat color QTL regions, are displayed in the Circos plot.

To further prioritize candidate *BnaMYBs* associated with seed coat color and oil content, previously reported seed coat color QTLs from multiple linkage populations and association mapping studies in *B. napus* were integrated and projected onto the Darmor-bzh v10 reference genome. A total of 41 consensus QTLs associated with seed coat color were collected and mapped onto 15 chromosomes (Figure 3). Among these chromosomes, A09 harbored the largest number of seed coat color QTLs (11), followed by C08 (6). Based on the physical intervals of these QTLs, 46 *R2R3-BnaMYBs* were identified within seed coat color-associated regions (Table S3). In addition, 169 oil content QTLs collected from published studies were mapped onto the Darmor-bzh v10 genome (Figure 3). These oil content QTLs exhibited a broad genomic distribution, with chromosome A09 harboring the largest number of oil content QTLs (24), followed by A03 (22). A total of 171 *R2R3-BnaMYBs* were identified within oil content-associated QTL regions (Table S4). The genomic positions of these R2R3-BnaMYBs were subsequently compared with the overlapping regions between seed coat color- and oil content-associated QTLs. Genes located within these shared QTL intervals were retained as candidate BnaMYBs, resulting in 32 R2R3-BnaMYBs potentially associated with both seed coat color and oil content.

After projection onto the Darmor-bzh v10 reference genome, seed coat color QTLs and oil content QTLs located on the same chromosome were considered co-localized when their physical intervals directly overlapped. R2R3-BnaMYBs located within these shared QTL intervals were then retained, resulting in a total of 32 candidate genes potentially associated with both seed coat color and oil content. Among these candidates, *BnaA09.MYB52* has previously been shown to affect both seed coat characteristics and seed oil content in *Brassica napus* [27]. In addition, *BnaC03.MYB56* has been implicated in seed oil accumulation in *B. napus* [28]. These previously characterized genes provide additional support for the biological relevance of the QTL-based candidate set [28].

### 2.4. Identification of Candidate BnaMYBs Associated with Proanthocyanidin Biosynthesis and Lipid Metabolism Through Integrative Co-Expression Analysis

Weighted gene co-expression network analysis (WGCNA) was performed using previous transcriptome data from four different tissues of seeds at two seed developmental stages of two varieties differing both in seed coat color and oil content [26]. A total of 15 co-expression modules were identified (Figure S3). Among these modules, the Blue3 module contained the majority of PA biosynthesis-related genes and numerous lipid biosynthesis- and metabolism-related genes. Therefore, this module was selected for further analysis based on its gene composition and functional relevance to these metabolic pathways. To focus on genes with reliable expression and strong co-expression relationships, genes in the Blue3 module were filtered using FPKM > 1.0 and edge weight > 0.25. After filtering, 1710 genes were retained for functional enrichment analysis. Gene Ontology (GO) and Kyoto Encyclopedia of Genes and Genomes (KEGG) enrichment analyses were performed using the BnIR database. The 1710 genes served as the query set, with the default genome-specific annotation provided by BnIR used as the reference background. Statistical significance was determined using multiple-testing-adjusted *p* values (p.adjust), with adjusted *p* < 0.05 considered significant.

**Figure 4:**
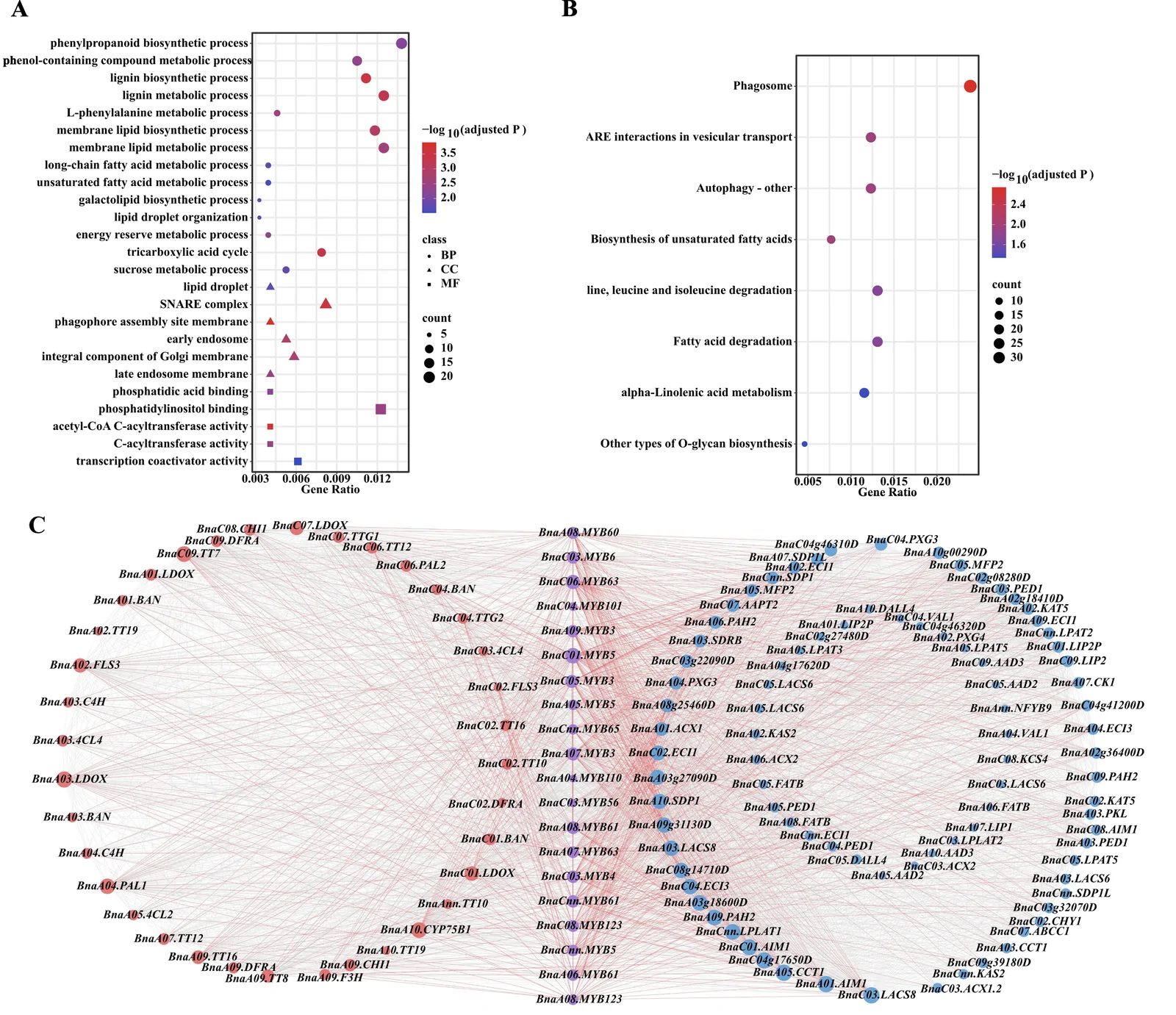
Functional enrichment analyses and co-expression network analysis of the Blue3 module for identification of candidate *BnaMYBs.* (**A**). GO enrichment analysis of the filtered genes in the Blue3 module. The *x*-axis represents the Gene Ratio, and the *y*-axis represents enriched GO terms. Circles, triangles, and squares denote Biological Process (BP), Cellular Component (CC), and Molecular Function (MF), respectively. Node size indicates the number of genes associated with each term (Count), and node color represents −log10(adjusted *p* value). Statistical significance was assessed using the adjusted *p* values reported by BnIR, with adjusted *p* < 0.05 considered significant. (**B**) KEGG pathway enrichment analysis of the filtered genes in the Blue3 module. The *x*-axis represents the Gene Ratio, and the *y*-axis represents selected KEGG pathways. Node size indicates the number of genes associated with each pathway (Count), and node color represents −log10(adjusted *p* value). Statistical significance was assessed using the adjusted *p* values reported by BnIR, with adjusted *p* < 0.05 considered significant. (**C**) Refined co-expression network of proanthocyanidin (PA) biosynthesis-related genes, lipid metabolism-related genes, and *BnaMYBs* in the Blue3 module. Only genes with FPKM > 1.0 and co-expression relationships with edge weights > 0.25 were retained in the network. Nodes represent genes, whereas edges represent co-expression relationships between genes. Red nodes represent PA biosynthesis-related genes, purple nodes represent BnaMYBs, and blue nodes represent lipid metabolism-related genes.

GO enrichment analysis based on multiple-testing-adjusted *p* values (adjusted *p* < 0.05) revealed significant enrichment of biological processes related to phenylpropanoid metabolism, phenol-containing compound metabolism, fatty acid metabolism, and lipid metabolic processes (Figure 4A). Specifically, the phenylpropanoid biosynthetic process, phenol-containing compound metabolic process, lignin biosynthetic process, and lignin metabolic process were significantly enriched, indicating a potential involvement of the Blue3 module in secondary metabolism associated with seed coat pigmentation. In addition, membrane lipid biosynthetic process, membrane lipid metabolic process, and fatty acid metabolic processes were enriched, suggesting potential associations with lipid metabolism during seed development. Among the KEGG pathways related to lipid metabolism, biosynthesis of unsaturated fatty acids, fatty acid degradation, and α-linolenic acid metabolism remained significantly enriched after multiple-testing correction (adjusted *p* < 0.05; Figure 4B). Other biologically relevant pathways represented in Figure 4B were not interpreted as statistically significant when their adjusted *p* values were ≥0.05. These results showed that the Blue3 module was enriched in genes related to both flavonoid/PA metabolism and lipid metabolism, suggesting a potential co-expression relationship between these metabolic processes during seed development.

To further identify *BnaMYBs* potentially associated with both proanthocyanidin (PA) biosynthesis and lipid metabolism, a refined co-expression network was constructed using the filtered genes from the Blue3 module. This network included PA biosynthesis-related genes, lipid metabolism-related genes, and *BnaMYBs* retained after expression and co-expression filtering (Figure 4C). Among these candidates, 20 *BnaMYBs* exhibited co-expression connections with both PA biosynthesis-related genes and lipid metabolism-related genes, suggesting their potential association with both metabolic processes.

Among these candidate genes, *BnaC05.MYB5* showed the highest connectivity within the refined co-expression network, followed by *BnaC06.MYB63*, *BnaA08.MYB61*, and *BnaA07.MYB63*. These highly connected *BnaMYBs* may represent candidate genes associated with PA metabolism and lipid-related processes during seed development.

In addition, several *BnaMYBs*, including *BnaA08.MYB60* and *BnaC04.MYB101*, displayed stronger associations with lipid metabolism-related genes but relatively fewer connections with PA biosynthesis-related genes, suggesting that these genes may be preferentially associated with seed lipid metabolism processes.

### 2.5. Co-Expression Network and Haplotype Analysis of Three Candidate BnaMYBs Associated with Seed Coat Pigmentation and Oil Accumulation

**Figure 5:**
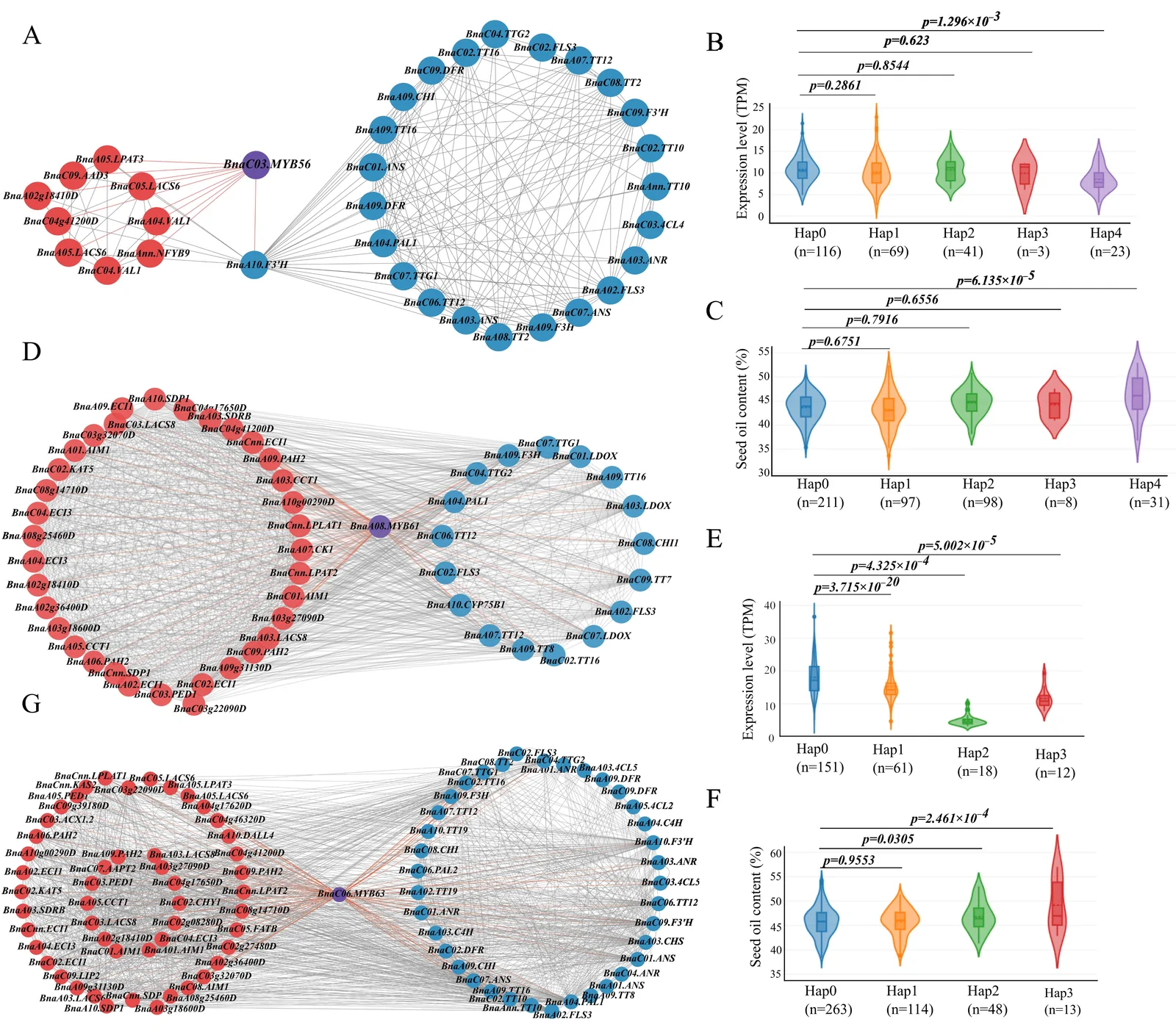
Local co-expression networks and haplotype analyses of three core BnaMYB candidate genes. (**A**) Local co-expression network of *BnaC03.MYB56*. The purple node represents *BnaC03.MYB56*, red nodes represent lipid metabolism-related genes, and blue nodes represent seed coat color/proanthocyanidin (PA) biosynthesis-related genes. Red edges indicate direct co-expression relationships with *BnaC03.MYB56*, whereas gray edges represent co-expression relationships among the remaining genes in the network. (**B**,**C**) Haplotype analysis of Bna*C03.MYB56*: (**B**) expression levels of *BnaC03.MYB56* in seeds at 40 days after flowering (40 DAF) among different haplotypes; (**C**) distribution of seed oil content among different haplotypes. (**D**) Local co-expression network of *BnaA08.MYB61*; node and edge colors are defined as in (**A**). (**E**,**F**) Haplotype analysis of *A08.MYB61*: (**E**) Expression levels of Bna*A08.MYB61* in seeds at 40 DAF among different haplotypes; (**F**) distribution of seed oil content among different haplotypes. (**G**) Local co-expression network of *BnaC06.MYB63*; node and edge colors are defined as in (**A**). In the violin plots, *n* indicates the number of accessions in each haplotype group, and *p* values represent the results of the overall statistical tests among haplotypes.

Based on the integration of expression patterns, QTL co-localization, co-expression evidence, and natural variation information, three BnaMYBs—*BnaC03.MYB56*, *BnaA08.MYB61*, and *BnaC06.MYB63*—were identified as candidate genes for further investigation. Haplotype analysis was subsequently performed to evaluate the relationship between natural variation at these loci and gene expression and seed oil content. To further characterize the co-expression patterns of the three core *BnaMYB* candidates and assess the relationship between their natural variation and seed oil content, local co-expression networks centered on *BnaC03.MYB56, BnaA08.MYB61*, and *BnaC06.MYB63* were extracted from the global co-expression network. These three candidates, located within QTL regions co-localized for seed coat color and oil content, were then subjected to haplotype analyses using natural population data (Figure 5). All three candidates showed co-expression relationships with proanthocyanidin (PA) biosynthesis-related genes and lipid metabolism-related genes, although their network connectivity patterns differed. Haplotype analysis identified multiple haplotypes for BnaC03.MYB56 and BnaA08.MYB61, allowing comparisons of gene expression and seed oil content among haplotype groups. In contrast, BnaC06.MYB63 showed limited haplotype diversity, with only one predominant haplotype detected in the analyzed population; therefore, a meaningful haplotype-based association analysis could not be performed for this gene. The haplotype composition, frequency distribution, and numbers of accessions are summarized in Table S5.

*BnaC03.MYB56* exhibited a relatively restricted local co-expression network. Among PA/flavonoid metabolism-related genes, *BnaC03.MYB56* showed a direct co-expression relationship mainly with the key structural gene *BnaF3′H*. In addition, several lipid metabolism-related genes, including *LPAT3*, *LACS6*, and *AAD3*, were also identified in its co-expression network (Figure 5A), suggesting that *BnaC03.MYB56* may be associated with the relationship between flavonoid metabolism and lipid accumulation during seed development. Five haplotypes of *BnaC03.MYB56* were identified in the natural population. Significant differences in *BnaC03.MYB56* expression levels were observed among different haplotypes (Figure 5B), accompanied by significant variation in seed oil content (Figure 5C). These results indicate that natural variation at the *BnaC03.MYB56* locus was significantly associated with variation in seed oil content, suggesting that this locus represents a potential candidate associated with oil accumulation during seed development.

*BnaA08.MYB61* displayed a broader local co-expression network. In the refined network, *BnaA08.MYB61* showed co-expression relationships with multiple PA/flavonoid metabolism-related genes, including *TTG1*, *TTG2*, *PAL1*, *TT12*, *FLS3*, *TT8*, *CHI1*, and *LDOX* (Figure 5D). These genes are important components of the flavonoid/proanthocyanidin pathway and have been implicated in seed coat pigmentation and PA accumulation. Moreover, *BnaA08.MYB61* was also connected with several lipid metabolism-related genes, including *LACS8*, *LPAT1*, *LPAT2*, *AIM1*, *PAH2*, *CCT1*, *ECI1*, and *ECI3*. The simultaneous association with PA biosynthesis-related and lipid metabolism-related genes suggests that *BnaA08.MYB61* may represent a potential candidate associated with flavonoid metabolism, seed coat development, and oil metabolism. Haplotype analysis revealed multiple natural variants of *BnaA08.MYB61*. Significant differences in transcript abundance were observed among different haplotypes (Figure 5E), which were also associated with significant variation in seed oil content (Figure 5F). These findings suggest that natural variation at the *BnaA08.MYB61* locus may influence gene expression and contribute to variation in seed oil accumulation.

*BnaC06.MYB63* also exhibited extensive co-expression relationships with both PA-related and lipid metabolism-related genes. In its local co-expression network, *BnaC06.MYB63* was associated with multiple PA/flavonoid metabolism-related genes, including *TT2*, *TTG2*, *CHS*, *F3′H*, *ANS*, *ANR*, *TT10*, and *TT12*, as well as several lipid metabolism-related genes (Figure 5G). However, unlike *BnaC03.MYB56* and *BnaA08.MYB61*, *BnaC06.MYB63* showed limited natural variation in the analyzed population, with only one predominant haplotype detected. This limited haplotype diversity suggests that genetic variation at the *BnaC06.MYB63* locus may be relatively conserved in the population.

## 3. Discussion

Proanthocyanidin (PA) biosynthesis is mainly regulated at the transcriptional level through coordinated expression of flavonoid biosynthetic genes [29]. Among the known regulators, R2R3-MYB transcription factors play central roles in controlling flavonoid and PA accumulation in plants. In *Arabidopsis thaliana*, *TT2 (MYB123)* was the first identified MYB regulator specifically involved in PA deposition in the seed coat, and homologous regulators have subsequently been characterized in *grapevine, Medicago truncatula, poplar*, and several crop species [30,31,32,33]. The close evolutionary and syntenic relationships between *Arabidopsis* and *Brassica *species provide a basis for identifying PA-related genes in *B. napus* through comparative genomics [34,35]. The progenitors of *B. rapa* and *B. oleracea* experienced an ancestral whole-genome triplication after their divergence from the *Arabidopsis* lineage, and their subsequent hybridization gave rise to allotetraploid *B. napus* [34,36,37]. Consequently, many functionally characterized *Arabidopsis* genes correspond to multiple co-orthologous copies in the A and C subgenomes of *B. napus*. We therefore used known PA-related genes from Arabidopsis as references to identify putative homologs in *B. napus*, and further prioritized candidate genes according to their expression patterns and co-expression relationships. In line with these genomic events, our genome-wide analysis revealed extensive expansion of the R2R3-MYB family in *B. napus*, reflecting the retention and potential functional diversification of duplicated genes following polyploidization [38,39].

Seed coat color and oil content are closely associated agronomic traits in *B. napus*, with yellow-seeded genotypes generally showing higher oil content than black-seeded genotypes [5]. Previous studies have shown that changes in TT2- and TT8-mediated flavonoid regulation are accompanied by alterations in seed pigmentation, fatty acid composition, and oil accumulation during seed development [6]. Flavonoid and fatty acid biosynthesis draw on common carbon resources, including malonyl-CoA, and changes in carbon allocation toward PA biosynthesis may therefore affect the substrate available for lipid accumulation [12,40]. Accordingly, the association between PA-related transcription factors and oil content does not necessarily indicate direct regulation of lipid biosynthesis but may partly result from competition for metabolic precursors and redistribution of carbon flux. In the present study, most PA biosynthetic genes together with numerous lipid metabolism-related genes were enriched in the same co-expression module identified by WGCNA, providing further transcriptional evidence that genes involved in flavonoid metabolism and lipid metabolism may share related expression patterns during seed development [41].

During seed development, MYB transcription factors play a central regulatory role, governing multiple critical processes from seed morphogenesis to storage reserve accumulation [19,42]. More recently, the R2R3-MYB transcription factor *BnC07MYB3a* was shown to directly repress the flavonoid biosynthetic genes *BnTT6* and *BnBAN*, and to participate in the regulation of PA accumulation and seed oil content in *B. napus* [4]. By integrating transcriptome profiling, WGCNA, and QTL co-localization, three candidate MYB genes, namely *BnaA08.MYB61*, *BnaC03.MYB56*, and *BnaC06.MYB63*, were identified as candidate genes associated with seed coat pigmentation and oil accumulation. Among these candidates, *BnaA08.MYB61* displayed extensive connectivity with PA/flavonoid biosynthesis-related genes, including *TTG1*, *TTG2*, *PAL1*, *TT12*, *FLS3*, *TT8*, *CHI1*, and *LDOX*. These genes are key components of the flavonoid/proanthocyanidin pathway and have been reported to participate in PA accumulation and seed coat pigmentation in *Arabidopsis thaliana* and *B. napus* [43]. In *Arabidopsis thaliana*, *MYB61* has been reported to play an important role in seed coat development. Loss-of-function analysis revealed that *AtMYB61* mutants exhibited defects in seed coat mucilage deposition and extrusion, indicating that *MYB61* contributes to the differentiation and maturation of seed coat epidermal cells [44]. In addition, *AtMYB61* was shown to associate with multiple biological processes through a small gene regulatory network, suggesting its potential involvement in coordinating developmental and metabolic pathways [45]. Although the biological function of *BnaA08.MYB61* in *B. napus* remains largely unexplored, its evolutionary conservation with *AtMYB61*, together with its location within co-localized QTL regions for seed coat color and oil content and its extensive connections with PA biosynthesis-related and lipid metabolism-related genes in our co-expression network, suggests that *BnaA08.MYB61* may represent a promising candidate associated with seed coat development, PA accumulation, and seed oil metabolism in rapeseed. Further functional validation will be required to determine its specific regulatory roles. *BnaC06.MYB63* exhibited strong co-expression relationships with both numerous PA biosynthetic genes and oil content-related genes. *MYB63* homologs have been widely reported as key candidate genes of lignin biosynthesis and secondary cell wall formation [46,47]. Previous studies demonstrated that lignin accumulation is closely associated with seed coat pigmentation in Brassica species, and several candidate genes related to lignin accumulation or seed color formation, including *BnCCR1* and *BnAHA10*, have been implicated in yellow-seeded traits [48]. Therefore, the extensive connectivity observed between *BnaC06.MYB63* and PA pathway genes suggests that this gene may represent a candidate associated with flavonoid metabolism and lignin-related processes during seed coat development. Changes in these secondary metabolic pathways may also influence carbon allocation during seed development, providing a possible explanation for the association of *BnaC06.MYB63* with oil content variation. However, whether *BnaC06.MYB63* directly associates with lipid metabolism remains to be experimentally determined.

Compared with *BnaA08.MYB61* and *BnaC06.MYB63, BnaC03.MYB56* displayed a smaller co-expression network and was primarily associated with *F3′H*., the key structure gene of proanthocyanin biosynthesis. The ortholog, *AtMYB56*, has been functionally characterized as a regulator of sucrose-mediated anthocyanin accumulation and flavonol biosynthesis in *Arabidopsis* [49]. Furthermore, *BnaC03.MYB56* resides within overlapping QTL intervals for both seed coat color and oil content, strengthening its candidacy. The haplotype analysis further revealed significant associations between natural variation at the *bnaC03.MYB56* locus and both gene expression level and seed oil content, suggesting that this gene may influence oil accumulation indirectly, possibly through metabolic crosstalk between carbohydrate and flavonoid pathways.

Collectively, our findings provide a comprehensive genomic resource for understanding the potential relationship between seed coat pigmentation and oil accumulation in *B. napus*. The integration of phylogenetics, transcriptomics, QTL mapping, and network analysis has proven effective in narrowing down candidate genes from a large gene family. Future functional characterization, including targeted mutagenesis and overexpression studies, will be essential to dissect the precise molecular mechanisms underlying the association between flavonoid and lipid pathways. The candidate genes identified here hold promising potential as targets for marker-assisted selection or gene editing to accelerate breeding of yellow-seeded, high-oil-content rapeseed cultivars.

## 4. Materials and Methods

### 4.1. Collection and Identification of Proanthocyanidin Biosynthesis, Lipid Metabolism-Related Genes and MYBs

The identification of PA biosynthesis-related genes in *B. napus* was performed based on syntenic and non-syntenic homologous relationships with *A. thaliana.* Functionally characterized flavonoid/PA biosynthesis-related genes from *A. thaliana* were used as reference genes. The *B. napus* Darmor-bzh v10 reference genome and annotation files were obtained from the *Brassica napus* multi-omics information resource BnIR (https://yanglab.hzau.edu.cn/BnIR, accessed on 11 May 2025). Candidate MYB domain-containing proteins were screened using the Pfam-A HMM database (https://ftp.ebi.ac.uk/pub/databases/Pfam/current_release/, accessed on 11 May 2025) through the HMM search function implemented in TBtools. A total of 1113 MYB domain-containing proteins were initially identified from the Darmor-bzh v10 proteome. The identified candidates were further validated using the NCBI Conserved Domain Database (CDD, https://www.ncbi.nlm.nih.gov/Structure/cdd/cdd.shtml, accessed on 11 May 2025). Proteins containing two adjacent MYB/SANT repeat regions, supported by at least three of the four core MYB-related domain models and lacking alternative conserved domains, were retained as high-confidence R2R3-MYB transcription factors.

Additionally, 126 R2R3-MYB transcription factor sequences from *A. thaliana* were downloaded from TAIR (http://www.Arabidopsis.org/, accessed on 11 May 2025). To correlate proanthocyanidin biosynthesis with seed oil-related pathways, oil content-related genes were collected from previously published studies and public databases (http://aralip.plantbiology.msu.edu/, accessed on 11 May 2025) [50,51]. Genes involved in seed oil accumulation, fatty acid biosynthesis, fatty acid elongation, fatty acid metabolism, biosynthesis of unsaturated fatty acids, triacylglycerol assembly, and lipid storage were included in this analysis. The corresponding homologous genes in *B. napus* were identified using the Darmor-bzh v10 reference genome and genome annotation files downloaded from BnIR (https://yanglab.hzau.edu.cn/BnIR, accessed on 11 May 2025).

Syntenic orthologs between *A. thaliana* and *B. napus* were identified using the *B. napus* pan-genome information resource (http://cbi.hzau.edu.cn/bnapus/, accessed on 11 May 2025), based on sequence similarity and the collinearity of flanking genes. The sequence similarity cutoff for syntenic ortholog identification was set to E ≤ 1 × 10^−20^. Non-syntenic homologs were further identified using BLASTN and BLASTP (BLAST+ v2.12.0, NCBI, Bethesda, MD, USA) searches with the gene and protein sequences of *A. thaliana* reference genes as queries. Candidate homologs were retained using an E-value cutoff of ≤1 × 10^−10^ and sequence coverage ≥ 0.75.

### 4.2. Chromosomal Localization, Synteny and Phylogenetic Analysis

The genomic annotation information of PA biosynthesis-related and MYB genes in *B. napus* was extracted from the *B. napus* Darmor-bzh v10 genome annotation file. The chromosomal locations of these genes were mapped using TBtools software v2.080 [52]. Collinearity analysis was performed using MCScanX v1.0 (University of Georgia, Athens, GA, USA) with the parameters gap_penalty = −1 and E-value = 1 × 10^−10^ as described previously [53]. Collinear gene pairs were identified based on sequence similarity and conserved gene order.

Multiple sequence alignment was performed using Clustal Omega v1.2.4 (EMBL-EBI, Hinxton, Cambridge, UK) based on conserved R2R3 MYB domain sequences [54]. The phylogenetic tree was constructed using MEGA11 based on the Maximum Likelihood method [55]. A majority-rule consensus tree was inferred from 1000 bootstrap replicates to evaluate the reliability of the tree topology.

### 4.3. Transcriptome Data Retrieval and Hierarchical Cluster and Enrichment Analysis

Transcriptome data of different seed tissue samples in *B. napus* were obtained from our previously published studies [26]. The RNA-seq data are available in the NCBI Sequence Read Archive (SRA) under accession number SRP469557. The dataset contained four seed tissues—the outer cotyledon (OC), inner cotyledon (IC), radicle (R) and seed coat (SC)—of yellow- and black-seeded materials at two seed developmental stages, at 24 and 33 days after flowering, with three biological [53] replicates for each condition. For visualization of gene expression patterns in the heatmaps, expression values from the three biological replicates were averaged for each condition. In contrast, biological replicates were retained as independent samples for WGCNA to preserve biological variation among samples. The processed FPKM expression matrix was used for subsequent expression analysis and weighted gene co-expression network analysis. Hierarchical clustering was performed based on gene expression levels using Euclidean distance as the distance metric and the complete-linkage method. Only genes (rows) were clustered, whereas samples (columns) were retained in their original input order without clustering.

Protein sequences of the *Brassica napus* Darmor-bzh v10 reference genome were downloaded from the BnaOmics database [56] and functionally annotated using eggNOG-mapper to obtain the corresponding Gene Ontology (GO) terms and KEGG Orthology (KO) identifiers [57]. GO and KEGG enrichment analyses were performed using the BnIR database. The 1710 genes in the Blue3 module were used as the query set, and the genome-specific annotation provided by BnIR was used as the reference background. Multiple-testing-adjusted *p* values (p.adjust) provided by BnIR were used to evaluate enrichment significance, and GO terms or KEGG pathways with adjusted *p* < 0.05 were considered significantly enriched. GO terms and KEGG pathways with adjusted *p* values < 0.05 were considered significantly enriched.

### 4.4. Co-Expression Network Analysis

Weighted gene co-expression network analysis (WGCNA) was conducted using the WGCNA package in R (v1.68). A soft-thresholding power of 14, a minimum module size of 30, and a merge Cut Height of 0.25 were applied for module identification.

The resulting modules were screened to identify those enriched in PA biosynthesis-related genes and lipid metabolism-related genes. Among the identified modules, the Blue3 module was selected for further analysis because it contained a relatively high proportion of PA biosynthesis-related and lipid metabolism-related genes. Module selection was therefore based on gene composition and functional enrichment. To retain genes with reliable expression and robust co-expression relationships, genes and edges within the Blue3 module were further filtered using thresholds of FPKM > 1.0 and edge weight > 0.25. After filtering, the refined co-expression network was visualized using the software Cytoscape 3.2 for candidate MYB screening [58].

### 4.5. QTL Physical Location Identification and Co-Localization Analysis

Previously reported QTLs associated with seed coat color and seed oil content in *Brassica napus* were collected from published studies [4,5,50,59,60]. To project these QTLs onto the *B. napus* Darmor-bzh v10 reference genome, primer sequences of flanking markers were aligned to the reference genome using BLASTN [61]. For PCR-based markers, such as simple sequence repeat (SSR), only primer pairs whose forward and reverse primers were located on the same chromosome and generated an expected physical interval of less than 500 bp were retained for further analysis. The physical interval of each QTL was determined according to the mapped positions of its flanking markers. Seed coat color QTLs and seed oil content QTLs were compared based on their physical intervals. Only QTL pairs showing direct physical interval overlap after projection onto the Darmor-bzh v10 genome were regarded as co-localized QTLs.

The chromosomal positions of *BnaMYB* genes were then compared with these co-localized QTL intervals, and *BnaMYB* genes located within the shared genomic regions of seed coat color and oil content QTLs were considered candidate genes [62]. For visualization, all *BnaMYB* genes located within both seed coat color QTLs and seed oil content QTLs were retained and highlighted in red, whereas only a subset of *BnaMYB* genes located within either seed coat color QTLs or seed oil content QTLs was displayed in black to improve figure readability.

### 4.6. Haplotype Analysis of Candidate Genes

Haplotype analysis of selected candidate *BnaMYBs* was performed in the BnIR database (https://yanglab.hzau.edu.cn/BnIR, accessed on 11 May 2025) using the available variation, gene expression, and seed oil content information. The corresponding BnIR gene IDs of the candidate *BnaMYBs* were used to retrieve haplotype groups, expression levels during seed development, and seed oil content data. Haplotypes represented by sufficient numbers of accessions were retained for comparison. Differences in gene expression and seed oil content among haplotypes were evaluated based on the statistical results provided by BnIR.

## 5. Conclusions

In summary, this study systematically identified 102 PA biosynthesis-related genes and 416 R2R3-MYB genes in the *B. napus* genome and provided insights into their expansion and functional diversification after polyploidization. Through integrative transcriptome profiling, WGCNA, and QTL co-localization analyses, we uncovered a major co-expression module (Blue3) that links PA biosynthesis with lipid metabolism, providing transcriptional evidence for the potential association between seed coat pigmentation and oil accumulation. Among the prioritized candidates, *BnaC03.MYB56*, *BnaA08.MYB61* and *BnaC06.MYB63* exhibited broad co-expression with multiple PA biosynthetic and lipid metabolism-related genes, suggesting that these genes represent candidate nodes associated with both pathways. Haplotype analyses further supported the association of natural variation at *BnaC03.MYB56* and *BnaA08.MYB61* loci with gene expression and seed oil content, whereas *BnaC06.MYB63* showed limited haplotype diversity. These findings provide insights into the genetic and transcriptional associations underlying seed coat pigmentation and oil accumulation in *B. napus* but also provide valuable candidate genes for molecular breeding and genetic improvement of yellow-seeded, high-oil-content rapeseed cultivars. Future functional validation through targeted mutagenesis and transgenic approaches will be essential to determine whether and how these MYB transcription factors contribute to the relationship between flavonoid and lipid pathways.

## Data Availability

The original contributions presented in this study are included in the article/Supplementary Materials. Further inquiries can be directed to the corresponding authors. Transcriptome data used in this study are available through the NCBI (SRP469557).

## Supplementary Materials

The following supporting information can be downloaded at https://www.mdpi.com/article/10.3390/ijms27209161/s1.
