# Supplementary material for: Identification of Candidate MYB Regulators of Seed Coat Color and Oil Content in Brassica napus
Source: Int J Mol Sci. 2026 Oct 15;27(20):9161. doi: 10.3390/ijms27209161 (PMC13705267; doi:10.3390/ijms27209161)
Supplement: Supplementary file 1 [file ijms-27-09161-s001.zip › supplementary figure caption.pdf]

Figure S1. Phylogenetic analysis of R2R3-MYB transcription factors in *Brassica napus*. An unrooted phylogenetic tree was constructed using the full-length protein sequences of *B. napus* R2R3-MYBs (BnaMYBs) containing the conserved MYB domain. The tree also includes R2R3-MYBs from *Arabidopsis thaliana* and functionally characterized proanthocyanidin (PA)-related MYBs from other plant species to infer evolutionary relationships. The BnaMYBs are classified into different subfamilies, indicated by different colored sectors on the outer ring. Branches are colored according to their respective clades.

Figure S2. Heatmap of *BnaMYB* gene expression during seed development. Hierarchical clustering was performed on eight samples from two varieties (K24 and K33). The x-axis labels indicate the specific tissues: SC, OC, IC, and R. Red and blue colors indicate up-regulated and down-regulated expression levels, respectively, according to the scale bar on the right. Distinct clusters of co-expressed genes are visible across the different tissue types.

Figure S3. Weighted gene co-expression network analysis (WGCNA) of seed transcriptomes. Gene clustering dendrogram (top) and corresponding module distribution (bottom) derived from two varieties differing in seed coat color and oil content. Genes were clustered into 15 distinct co-expression modules based on their expression patterns across four tissues at two developmental stages. Different colors in the ''Module'' row indicate different gene modules.
